# Supplementary material for: Within-Country Inequality in COVID-19 Vaccination Coverage: A Scoping Review of Academic Literature
Source: Vaccines (Basel). 2023 Feb 23;11(3):517. doi: 10.3390/vaccines11030517 (PMC10058740; doi:10.3390/vaccines11030517)
Supplement: Supplementary file 1 [file vaccines-11-00517-s001.zip › vaccines-2230177-supplementary.pdf]

S1. Scoping review protocol

Note: this protocol was developed in adherence with the JBI Manual for Evidence Synthesis [1]. Modifications and clarifications made to the initial protocol are indicated in red text.

Background: The COVID-19 pandemic restrictions and responses have exacerbated many forms of social inequalities, which may also be evident in COVID-19 immunization. There is a growing body of research exploring the nature of COVID-19 immunization inequality across diverse settings, with relevant lessons and insights for strengthening equity in vaccine policies and planning. A review of this body of literature is necessary to characterize the types of immunization inequalities measured in these studies (including the dimensions of inequality) and compile preliminary evidence about the patterns of inequality and areas for further inquiry. The findings of the proposed review will be useful to inform the development of further research on the topic of inequalities in COVID-19 immunization, including secondary analyses of global datasets, and systematic literature reviews and meta-analyses.

Published reviews on similar topics:

- A systematic review by Bayati et al (2022) assessed studies on inequalities in COVID-19 vaccine distribution [2]. It grouped findings as macro level factors (explaining inequalities between countries or regions) or micro level factors (explaining inequalities between individuals or households). The study concluded: "among micro level determinants, age, race, ethnic, household income, residency in the deprived areas, employment, poverty, location (urban/rural) and gender were most often mentioned in the literature." Due to a narrow search strategy, the review covered a limited number of studies addressing micro level factors (n=19) and, per the scope of the review, did not discuss in detail the nature of the findings and their implications.
- Robinson et al (2021) conducted a systematic review and meta-analysis of evidence about the intended uptake and refusal of COVID-19 vaccines [3]. The study was conducted prior to the mass rollout of the vaccine and is based on 20 articles pertaining to nationally representative populations in 13 countries. Findings suggested that, in addition to overall declining intention to vaccine throughout the course of the pandemic, "being female, younger, of lower income or education level and belonging to an ethnic minority group are associated with a reduced likelihood of intending to be vaccinated when a vaccine becomes available". The article concludes by pointing to "an urgent need to address social inequalities in vaccine hesitancy and promote widespread uptake of vaccines as they become available" and therefore provides support for carrying out the proposed review.

Central question: what is the current status of literature on within-country inequalities in COVID-19 vaccination coverage?

Review objectives: The objectives of the paper are to (a) describe how within-country inequalities in COVID-19 vaccination are characterized in the academic literature, (b) describe trends in the reported extent and directionality of inequalities, and (c) provide an overview of the gaps and future recommendations for research.

Scoping review methods

Justification

The proposed paper will be a scoping review. Scoping reviews are appropriate “to determine the scope or coverage of a body of literature on a given topic and give clear indication of the volume of literature and studies available as well as an overview (broad or detailed) of its focus. Scoping reviews are useful for examining emerging evidence when it is still unclear what other, more specific questions can be posed and valuably addressed by a more precise systematic review” [4].

Inclusion and exclusion criteria

1. **Population**. The study must pertain to human populations.
2. **Excluded article types**. The following article types will be excluded: comments, letters, editorials, conference proceedings, news articles, biographies, reference materials, interviews. Pre-prints that are not peer reviewed will be excluded.
3. **Included article types.** Research articles and research reports (including systematic and other reviews) reporting primary or secondary data will be considered for inclusion. Pre-prints will be considered, but only if they have undergone peer review. Brief reports, short communications and short research articles (5 pages or less) will be considered for inclusion; if included, these types of articles are marked as “short research papers”.
4. **Study objective.** Articles will be considered for inclusion if they contain a primary or secondary study objective pertaining to reporting inequalities in COVID-19 vaccination coverage, which encompasses receipt or non-receipt of one or more COVID-19 vaccine doses and/or boosters. Studies were not included on the basis of a study objective to understand the drivers of inequalities.
5. **COVID-19 vaccination.** Articles will be considered for inclusion if COVID-19 vaccination coverage is defined as the receipt or non-receipt of a COVID-19 vaccine or booster. Articles with a study objective focused on (or encompassing) the following aspects of COVID-19 vaccination will be excluded:
   - intention to vaccinate
   - attitudes about vaccination
   - vaccine availability, accessibility and eligibility
   - access to vaccination sites
   - vaccine readiness
   - vaccine decision making factors
   - participation in vaccination trials
   - strategies for increasing vaccination uptake
   - predictive modelling of vaccination uptake
   - perceptions about vaccination uptake

   In other words, if the study definition of COVID-19 vaccination coverage included any of the above, it will be excluded.

6. **Dimension of inequality.** Articles will be considered for inclusion if they report vaccination coverage by one or more socioeconomic, demographic or geographic dimensions of inequality. Articles will be excluded if they focused on reporting vaccination coverage according to: medical factors; or the presence/absence of large employers.

7. **Level of measurement.** Articles will be considered for inclusion if dimensions of inequality are measured at the individual, household, community level or small area level. Articles with a study objective focused on reporting between-country inequalities will be excluded.
8. **Full text availability.** For inclusion, the full text of the article must be available.

Search strategy

Literature searches will be conducted systematically in PubMed, Scopus and Web of Science, with additional sources obtained through handsearching reference lists. No language, article type, or date restrictions will be applied (though due to the nature of the research question, the article would need to be published since the rollout of COVID-19 vaccines in late 2020). The search strategy will consist of three domains related to 'inequality' AND 'COVID-19' AND 'vaccines' search terms[1].

Study selection methods

The results from the literature search will be imported to Covidence software. Title and abstract scan will be conducted by one researcher, Nicole Bergen (NB). This will be followed by full text review by two reviewers, NB and Nicole Johns (NJ), in consultation with Ahmad Reza Hosseinpoor (ARH), as needed to reach agreement for inclusion/exclusion. For studies excluded during the full text review, the first reason for exclusion will be recorded, according to the following ordered list: (1) wrong article type; (2) does not pertain to humans; (3) study objective not relevant; (4) does not meet criteria for COVID-19 vaccine coverage; (5) does not meet criteria for dimension of inequality; (6) only reports on between-country inequality; (7) full text not available; (8) insufficient information to assess eligibility.

Data extraction and synthesis

A data extraction template will be applied to extract relevant information from each included article. This includes: general information about the article and where it was published; characteristics of the study setting, population, study objective and design; characteristics of the COVID-19 vaccination indicator; characteristics of the dimensions of inequality; analysis methods; results; and conclusions.

To the extent possible, the information will be copied from the original source into the data extraction template, and then tabulated across studies, noting where any terminology or concepts may be combined or adapted (e.g. tabulating dimensions of inequality addressing 'economic status' which may encompass different measurement approaches). After reviewing 10% of studies in tandem to ensure consistency in how the template is interpreted and applied, one researcher (NB or NJ) will do the data extraction for each included study. The researchers will periodically consult and reach consensus on any questions or points of ambiguity that arise.

Timeframe

Protocol established: Sept-Oct 2022

Search conducted: Oct 2022

---

[1]In the case of PubMed, additional searches will be conducted using MESH terms related to 'COVID-19 vaccines' (combined with the inequality domain using the AND operator).

Title and abstract screening: Oct 2022

Full text review and data extraction: Nov-Dec 2022

Write up: Dec 2022-Jan 2023

References

1. Peters, M.D.J.; Godfrey, C.; McInerney, P.; Munn, Z.; Tricco, A.C.; Khalil, H. Chapter 11—Scoping Reviews. In *JBI Manual for Evidence Synthesis*; Aromataris, E., Munn, Z., Eds.; JBI: North Adelaide, Australia, 2020.
2. Bayati, M.; Noroozi, R.; Ghanbari-Jahromi, M.; Jalali, F.S. Inequality in the Distribution of COVID-19 Vaccine: A Systematic Review. *Int. J. Equity Health* **2022**, *21*, 122. https://doi.org/10.1186/s12939-022-01729-x.
3. Robinson, E.; Jones, A.; Lesser, I.; Daly, M. International Estimates of Intended Uptake and Refusal of COVID-19 Vaccines: A Rapid Systematic Review and Meta-Analysis of Large Nationally Representative Samples. *Vaccine* **2021**, *39*, 2024–2034. https://doi.org/10.1016/j.vaccine.2021.02.005.
4. Munn, Z.; Peters, M.D.J.; Stern, C.; Tufanaru, C.; McArthur, A.; Aromataris, E. Systematic Review or Scoping Review? Guidance for Authors When Choosing between a Systematic or Scoping Review Approach. *BMC Med. Res. Methodol.* **2018**, *18*, 143. https://doi.org/10.1186/s12874-018-0611-x.

S2. Search terms

PubMed

(("Health Equity"[MeSH Terms] OR "Healthcare Disparities"[MeSH Terms] OR "Socioeconomic Factors"[MeSH Terms] OR "Health Services Accessibility"[MeSH Terms] OR "Social Determinants of Health"[MeSH Terms] OR "Social Discrimination"[MeSH Terms] OR "Right to Health"[MeSH Terms] OR "Epidemiologic Measurements"[MeSH Terms] OR "Social Environment"[MeSH Terms] OR "Population Groups"[MeSH Terms]) AND ((("COVID-19"[MeSH Major Topic] OR "covid 19/epidemiology"[MeSH Terms] OR "covid 19/prevention and control"[MeSH Terms] OR "covid 19/statistics and numerical data"[MeSH Terms]) AND ("Vaccination Coverage"[MeSH Major Topic] OR "vaccination coverage/statistics and numerical data"[MeSH Terms] OR "Vaccination"[MeSH Major Topic] OR "vaccination/epidemiology"[MeSH Terms] OR "vaccination/statistics and numerical data"[MeSH Terms] OR "vaccination/supply and distribution"[MeSH Terms] OR "vaccination/trends"[MeSH Terms])) OR "COVID-19 Vaccines"[MeSH Terms])) OR (("equit*"[Title/Abstract] OR "inequit*"[Title/Abstract] OR "inequal*"[Title/Abstract] OR "equal*"[Title/Abstract] OR "disparit*"[Title/Abstract] OR "socioeconomic factor*"[Title/Abstract] OR "social factor*"[Title/Abstract] OR "social determinant*"[Title/Abstract]) AND ("covid*"[Title/Abstract] OR "Coronavirus"[Title/Abstract] OR "sars cov*"[Title/Abstract] OR "sars cov*"[Title/Abstract] OR "2019 ncov"[Title/Abstract] OR "2019 ncov"[Title/Abstract]) AND ("vaccin*"[Title/Abstract] OR "immuniz*"[Title/Abstract] OR "immunis*"[Title/Abstract]))

Web of Science

((TS=equit*) OR (TS=inequit*) OR (TS=inequal*) OR (TS=equal*) OR (TS=disparit*) OR (TS=socioeconomic factor*) OR (TS=social factor*) OR (TS=social determinant*)) AND ((TS=COVID*) OR (TS=Coronavirus) OR (TS=SARS CoV*) OR (TS=SARS-CoV*) OR (TS=2019-nCoV) OR (TS=2019 nCoV)) AND (TS=Vaccin*) OR (TS=Immuniz*) OR (TS=Immunis*)

Scopus

(TITLE-ABS-KEY (vaccin*) OR TITLE-ABS-KEY (immuniz*) OR TITLE-ABS-KEY (immunis*)) AND (TITLE-ABS-KEY (covid*) OR TITLE-ABS-KEY (coronavirus) OR TITLE-ABS-KEY (sars AND cov*) OR TITLE-ABS-KEY (sars-cov*) OR TITLE-ABS-KEY (2019-ncov) OR TITLE-ABS-KEY (2019 ncov)) AND (TITLE-ABS-KEY (inequit*) OR TITLE-ABS-KEY (equit*) OR TITLE-ABS-KEY (equit*) OR TITLE-ABS-KEY (inequit*) OR TITLE-ABS-KEY (inequal*) OR TITLE-ABS-KEY (equal*) OR TITLE-ABS-KEY (disparit*) OR TITLE-ABS-KEY (socioeconomic AND factor*) OR TITLE-ABS-KEY (social AND factor*) OR TITLE-ABS-KEY (social AND determinant*))

S3. Study sources

Table S1. Number of articles included in a scoping review about inequalities in COVID-19 vaccination coverage, by journals where published

Number of articles	Journal(s) where published
18	Vaccines
15	Morbidity and Mortality Weekly Report
11	Vaccine
7	International Journal of Environmental Research and Public Health; PLoS One
5	BMJ Open
4	JAMA Network Open
3	American Journal of Infection Control; American Journal of Preventive Medicine; BMC Public Health; Emerging Infectious Diseases; Frontiers in Public Health; Health Psychology; Journal of Public Health
2	American Journal of Industrial Medicine; Clinical Infectious Diseases; European Journal of Public Health; Journal of Clinical Medicine; Journal of General Internal Medicine; Journal of Racial and Ethnic Health Disparities; Nature Communications; PLoS Medicine; Public Health Reports; Social Science and Medicine - Population Health; The Lancet Regional Health - Europe
1	American Journal of Epidemiology; American Journal of Obstetrics & Gynecology; American Journal of Tropical Medicine and Hygiene; Annals of Medicine; Australian and New Zealand Journal of Obstetrics and Gynaecology; BMC Geriatrics; BMC Infectious Diseases; Bratislava Medical Journal; Canadian Medical Association Journal; Clinical Microbiology and Infection; Communicable Diseases Intelligence; Conflict and Health; Expert Review of Vaccines; Harm Reduction Journal; Health Affairs; Health Psychology and Behavioral Medicine; Health Risk Analysis; Health Science Reports; Human Vaccines & Immunotherapeutics; Infectious Diseases of Poverty; Infection and Drug Resistance; Infection Control & Hospital Epidemiology; International Health; International Journal for Equity in Health; International Journal of Epidemiology; Israel Journal of Health Policy Research; Journal of Epidemiology and Community Health; Journal of Preventive Medicine; Journal of Urban Health; JMIR Public Health and Surveillance; Journal of Health Care for the Poor and Underserved; Journal of Immigrant and Minority Health; Journal of Infection and Public Health; Journal of Multidisciplinary Healthcare; Journal of Occupational Environmental Medicine; Journal of Personalized Medicine; Journal of Public Health and Development; Journal of Public Health Management and Practice; Journal of the American Geriatrics Society; Journal of Transcultural Nursing; Lancet; Medical Surveillance Monthly Report; Medicina; Military Medicine; Open Forum Infectious Diseases; Postgraduate Medical Journal; Prehospital Emergency Care; Preventing Chronic Disease; Preventive Medicine; Preventive Medicine Reports; Public Health; Scientific Reports; Socius; The Journal of Maternal-Fetal & Neonatal Medicine; The Lancet Regional Health - Americas; The Lancet Regional Health - Western Pacific; The Primary Care Companion for CNS Disorders
